# Monoclonal-Based Antivenomics Reveals Conserved Neutralizing Epitopes in Type I PLA_2_ Molecules from Coral Snakes

**DOI:** 10.3390/toxins15010015

**Published:** 2022-12-26

**Authors:** Carlos Corrêa-Netto, Marcelo A. Strauch, Marcos Monteiro-Machado, Ricardo Teixeira-Araújo, Juliana Guzzo Fonseca, Moema Leitão-Araújo, Maria Lúcia Machado-Alves, Libia Sanz, Juan J. Calvete, Paulo A. Melo, Russolina Benedeta Zingali

**Affiliations:** 1 Instituto Vital Brazil, Rio de Janeiro 24230-410, RJ, Brazil; 2 Instituto de Bioquímica Médica Leopoldo de Meis, Instituto Nacional de Biologia Estrutural e Bioimagem, Universidade Federal do Rio de Janeiro, Rio de Janeiro 21941-902, RJ, Brazil; 3 Programa de Farmacologia e Química Medicinal-UFRJ, Instituto de Ciências Biomédicas, Centro de Ciências da Saúde, Universidade Federal do Rio de Janeiro, Av. Carlos Chagas Filho, 373, Rio de Janeiro 21941-902, RJ, Brazil; 4 Fundação Zoobotânica do Rio Grande do Sul, Museu de Ciências Naturais, Núcleo Regional de Ofiologia de Porto Alegre, Porto Alegre 90690-000, RS, Brazil; 5 Laboratorio de Venómica Estructural y Funcional, Instituto de Biomedicina de Valencia, 46010 Valencia, Spain

**Keywords:** phospholipase A2, monoclonal antibody, neutralization PLA_2_ activity, enzymatic activity, myotoxicity

## Abstract

For over a century, polyclonal antibodies have been used to treat snakebite envenoming and are still considered by the WHO as the only scientifically validated treatment for snakebites. Nevertheless, moderate innovations have been introduced to this immunotherapy. New strategies and approaches to understanding how antibodies recognize and neutralize snake toxins represent a challenge for next-generation antivenoms. The neurotoxic activity of *Micrurus* venom is mainly due to two distinct protein families, three-finger toxins (3FTx) and phospholipases A_2_ (PLA_2_). Structural conservation among protein family members may represent an opportunity to generate neutralizing monoclonal antibodies (mAbs) against family-conserved epitopes. In this work, we sought to produce a set of monoclonal antibodies against the most toxic components of *M. altirostris* venom. To this end, the crude venom was fractionated, and its major toxic proteins were identified and used to generate a panel of five mAbs. The specificity of these mAbs was characterized by ELISA and antivenomics approaches. Two of the generated mAbs recognized PLA_2_ epitopes. They inhibited PLA_2_ catalytic activity and showed paraspecific neutralization against the myotoxicity from the lethal effect of *Micrurus* and *Naja* venoms’ PLA_2s_. Epitope conservation among venom PLA_2_ molecules suggests the possibility of generating pan-PLA_2_ neutralizing antibodies.

## 1. Introduction

Coral snakes are a large monophyletic group of over 80 small- to medium-sized colorful species of the Elapid family. Notable for their colorful red, yellow/white, and black colored banding, basal coral snake lineages originated in Asia [1,2] and are currently represented by two genera in the Old World (*Calliophis* and *Sinomicrurus*) and by three (*Leptomicrurus, Micruroides*, and *Micrurus*) in the New World [1,3,4]. However, separating *Micrurus* from *Leptomicrurus* is not a scientific consensus [3,5]. Extant New World coral snakes are widely distributed in the southern range of many temperate U.S. states, throughout Central America, and most of South America to central Argentina [6].

Envenomation by New World coral snakes is characterized by local manifestations, including myonecrosis [7,8], cardiovascular effects [9], and predominantly systemic neurotoxicity leading to respiratory arrest and death in severe cases [10,11,12]. A number of micrurine species have their venoms analyzed by venomic and transcriptomic approaches, including those from *M. surinamensis* [13,14]; *M. corallinus* [14,15,16]; *M. altirostris* [16]; *M. nigrocinctus* [17]; *M. mipartitus* [18]; *M. frontalis*, *M. ibiboboca*, *M. lemniscatus*, and *M. spixii*, [14,19,20,21]; *M. tener tener* [22]; *M. laticollaris* [23]; *M. fulvius* [24,25]; *M. mosquitensis* and *M. alleni* [26]; *M. dumerilii* [27]; *M. tschudii* [28]; *M. clarki* [29]; *M. pyrrhocryptus* [30]; *M. ruatanus* [31]; *M. browni browni* [32]; and more recently *M. yatesi* [33]. These studies have revealed that post-synaptic α-neurotoxins of the three-finger toxins (3FTx) family [34] and pre-synaptic phospholipase A_2_ (PLA_2_) molecules [35,36,37] represent the main toxin classes of *Micrurus* venoms. However, the relative abundance of these toxins varies widely between species [38]. Venomics combined with protein biochemical characterizations has contributed to a deeper understanding of the molecular determinants of the clinical effects of coral snake envenomation [22,23,32,39,40].

The Brazilian commercial Elapidic antivenom is produced at Butantan Institute (São Paulo) and Ezequiel Dias Foundation (Minas Gerais) by hyper-immunization of horses with equal amounts of venom from *M. corallinus* and *M. frontalis* [41], two snake species endemic to the south and southeast regions of Brazil. It has been reported that this antivenom is inefficient in fully neutralizing the neurotoxicity and lethality of some heterologous *Micrurus* species present in different geographic regions of the country [42,43,44,45]. An interesting aspect of some *Micrurus* venoms, especially those from *M. lemniscatus* and *M. altirostris*, is that despite having suitable antigens inducing a satisfactory immune response in horses, the generated antibodies have poor neutralizing capacity [44]. Additionally, the Brazilian commercial antivenom is ineffective in neutralizing lethality. In the case of *M. altirostris,* it has been demonstrated that its venom departs from others in immunochemical profile, biological activities, and toxin composition [16,42,44,45,46]. This species is distributed in southern Brazil, Uruguay, northeast Argentina, and Paraguay [47].

New strategies and immunization approaches are thus needed to generate improved antivenoms with a broader clinical usefulness landscape. In this regard, lethality and neurotoxicity among *Micrurus* species can be ascribed to just a few toxins, mainly PLA_2_ and 3FTx families [13,25,39,40]. This offers the possibility of generating an antivenom comprising a restricted set of anti-PLA_2_ and anti-3FTx neutralizing antibodies, like those already developed against certain scorpion and snake venoms [48,49,50]. As a proof-of-concept and the first step towards this goal, we have produced and analyzed the immunoreactivity of a set of monoclonal antibodies against the most toxic components of *M. altirostris* venom. Two PLA_2_-specific monoclonal antibodies cross-reacted with all the PLA_2_ molecules from *M. altirostris* venom, inhibiting their catalytic activity and myotoxicity. They also exhibited paraspecificity against PLA_2_s from *Naja naja* venom, demonstrating the conservation of paraspecific neutralizing epitopes across the Elapid family.

## 2. Results

### 2.1. Identification of the Major Toxic Components of M. altirostris Venom

**Figure 1:**
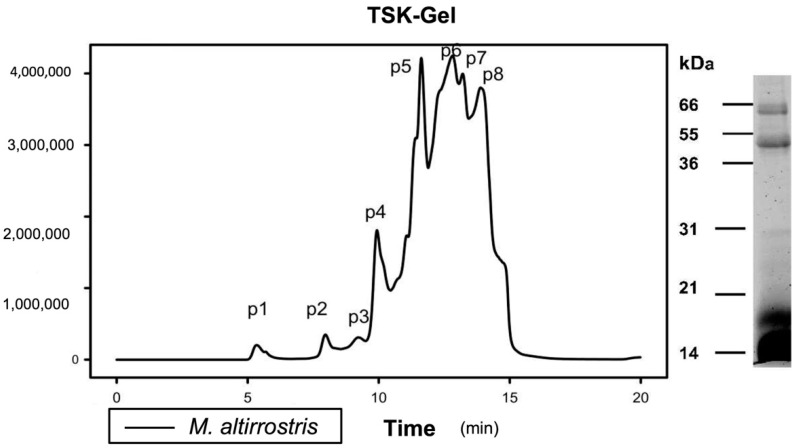
*M. altirostris* venom fractionation by gel filtration. The crude venom (20 mg) was submitted on TSK^®^-gel filtration. On the right side, an SDS-PAGE (15%) of the same venom was carried out under reducing conditions.

The toxicity of *M. altirostris* was analyzed using a toxicovenomics approach [51]. Briefly, the venom was fractionated by size-exclusion chromatography and measuring the LD_50_ of each manually collected fraction (Figure 1). Eight fractions were collected, and doses up to 8 μg/20 g per mouse were injected. Four fractions, P4, P6, P7, and P8, caused lethal effects, with LD_50_ of 3.3, 6.92, 7.07, and 2.97 μg per animal, respectively (Table 1).

The toxins present in these lethal fractions were identified by comparing their reverse-phase chromatographic profile with the whole venom run under the same conditions of the proteome previously characterized and reported in [16]. Table 1 shows the correlation between fractions from size exclusion and reverse-phase chromatographic separations. P4 comprised mainly PLA_2_ molecules; P6 and P7 contained 3FTxs and less than 6.4% of PLA_2_ molecules; and P8 was constituted of 3FTxs (Figures S1 and S2).

### 2.2. Monoclonal-Based Antivenomics Analyses of Antibodies against M. altirostris Venom Toxins

Size-exclusion chromatography of fraction P5 showed that *M. altirostris* venom contains roughly equal amounts of 3FTx and PLA_2_ molecules and is a less toxic fraction. This fraction was selected for mice immunization and subsequent generation of five stable hybridoma cells. Three monoclonal antibodies were specific for 3FTx and two for PLA_2_ (Table 2).

**Figure 2:**
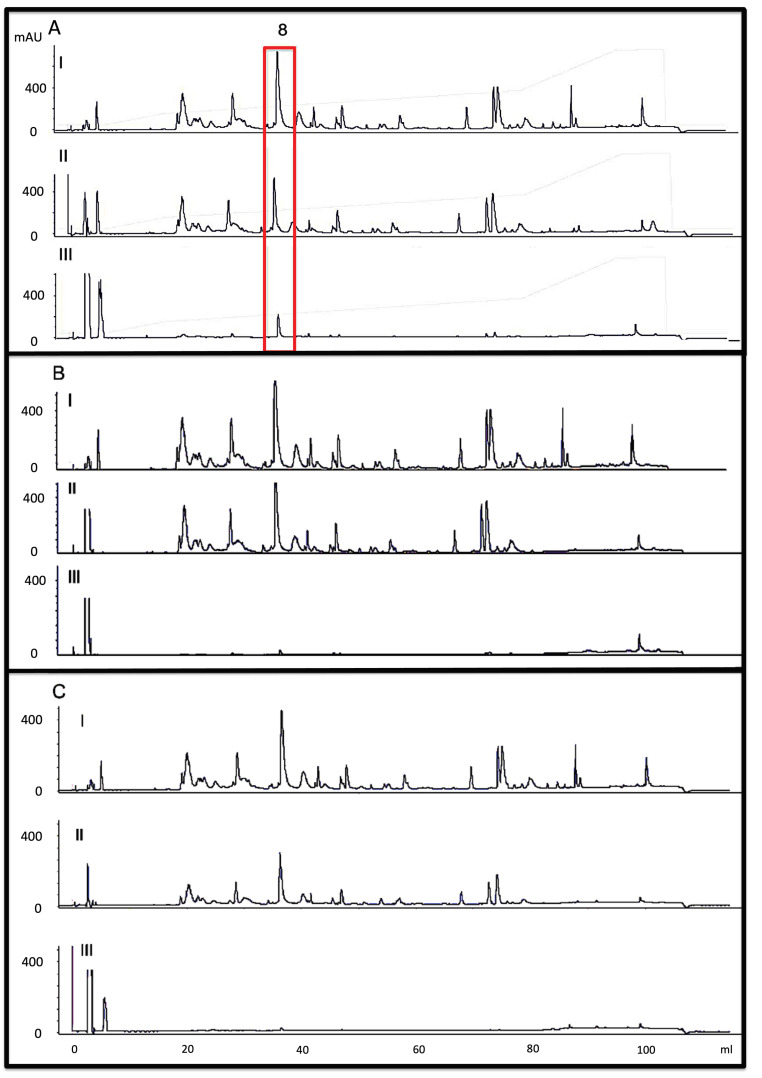

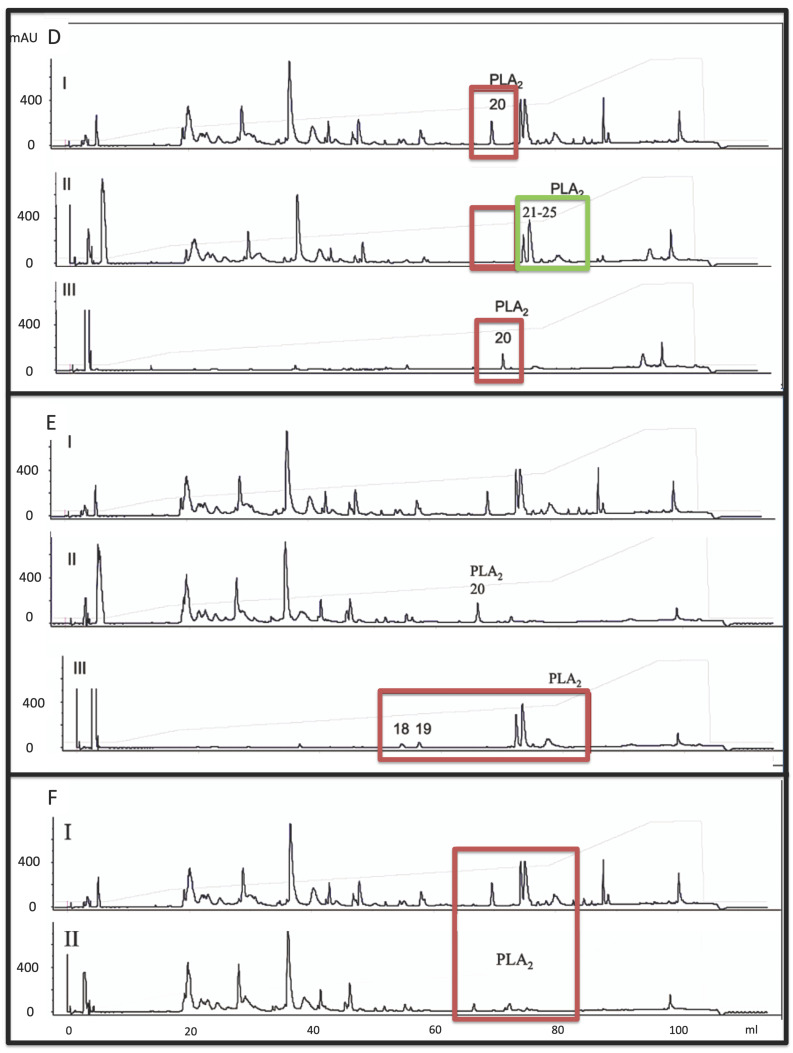
Monoclonal-based antivenomics. Five monoclonal antibodies (mAb) were analyzed in these experiments. (**A**) shows the results to mAb 4B3, (**B**) to mAb 2G2, (**C**) to mAb 2B, (**D**) to mAb 3B2, (**E**) to mAb 1E8, and Panel (**F**) to the association of mAb 1E8 and 3B2. The RP-HPLC separation of *M. altirostris* venom (2 mg) is demonstrated in (**A**–**I**), and this chromatogram was used for comparison with (**B**–**F**) experiments. The non-bound fractions from monoclonal antibodies’ affinity columns were analyzed in A-II, B-II, C-II, D-II, E-II, and F-II. The reverse-phase separations of the monoclonal-immunocaptured proteins are demonstrated in A-III, B-III, C-III, D-III, and E-III. Red boxes indicate peaks retained by the antibodies. Green box indicate other PLA2 peaks not retained by 3B2 mAB.

The identification of those toxins exhibiting toxin-family-specific epitopes recognized by mAbs was assessed by affinity chromatography-based antivenomics. Regarding the mAbs recognizing 3FTx (2G2, 2B1, and 4B3), our results revealed that only 4B3 showed specificity for the toxin present at peak 8 (P8), which has a high similarity to postsynaptic type-I α-neurotoxin (transcript MALT0059) with a molecular mass of 6688.2 Da (Figure 2A). Furthermore, 3FTxs displayed a high diversity of proteoforms, the mAbs lacked a broad binding capacity into this toxin family, or showed poor affinity (Figure 2B,C), hindering them for further characterization. On the other hand, monoclonal antibodies exhibited paraspecific binding towards PLA_2_. All PLA_2_ molecules from the venom of *M. altirostris* were recognized by the pair of monoclonal antibodies secreted by clones 3B2 and 1E8. Specifically, mAb 3B2 identified one proteoform eluted in fraction 20 of RP-HPLC (Figure 2D), the PLA_2_ molecule encoded by transcript F5CPF0 [16]. Except for fraction 20 (Figure 2E) mAb, 1E8 showed a broad binding capacity recognizing all PLA_2_ molecules, including the transcripts F5CPF1 and AED89576. The paraspecific complementary binding was further investigated to ascertain possible neutralization of the enzymatic and toxic activities of PLA_2_s.

### 2.3. Neutralization Properties of Monoclonal Antibodies towards PLA_2_ Toxicity

**Figure 3:**
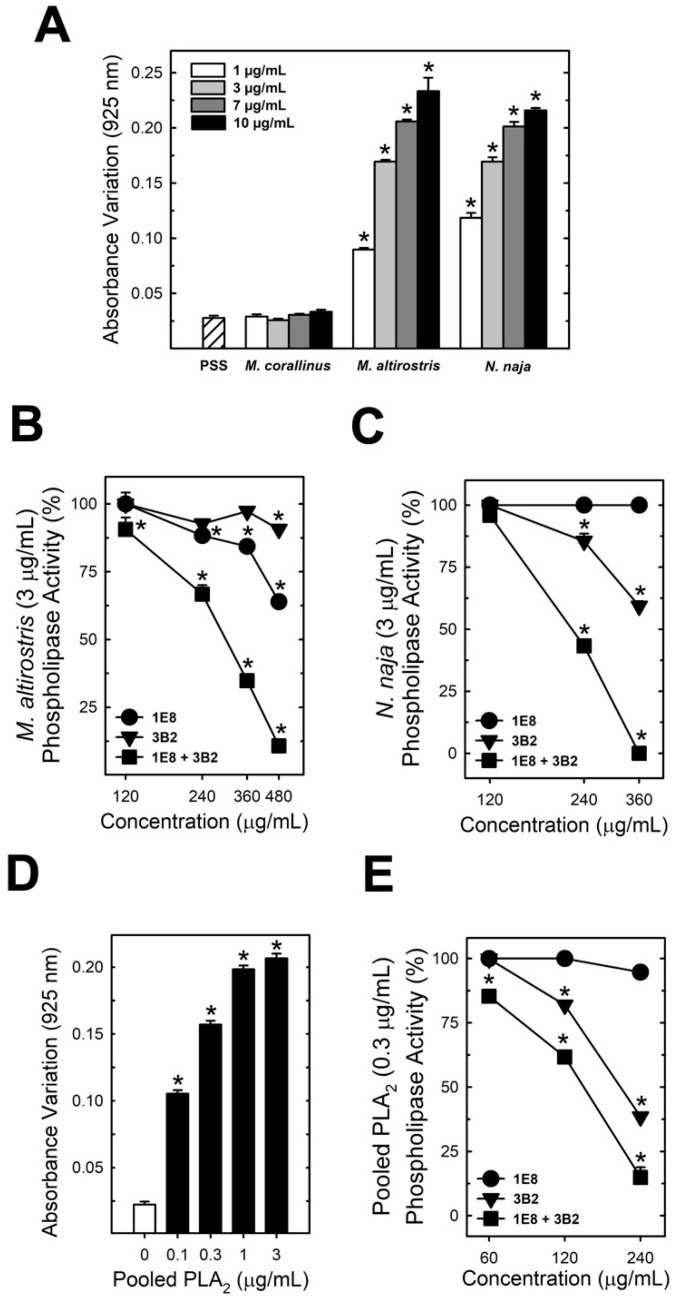
Effect of mAbs on the PLA_2_ enzymatic activity. Effect of immunoglobulins from clones 1E8 and 3B2 toward phospholipase activity. (**A**) shows the absorbance variation promoted by *M. corallinus*, *M. altirostris*, and *Naja naja* snakes (1–10 µg/mL) in an egg yolk solution (*n* = 8). (**B**,**C**) display respectively, the inhibition of *M. altirostris* (3 µg/mL) and *N. naja* venom (3 µg/mL) phospholipase activity by two immunoglobulins from clones 1E8 and 3B2 alone or as a mixture. (**D**), the change in the absorbance of egg yolk solution promoted by pooled PLA2 (P4) is shown. (**E**) displays the inhibition of pooled PLA_2_ (P4—0.3 µg/mL) by the immunoglobulins from clones 1E8 and 3B2 alone or combined. Data express mean ± SEM. (*n* = 8) * *p* < 0.05 vs. venom (**B**,**C**) or pooled PLA_2_ (**E**). Filled squares represented on (**B**,**C**,**E**) indicate that each mAb was combined according to the concentration indicated in the abscissa axis.

The ability of the mAbs to neutralize PLA_2_ catalytic activity was tested for the venom of *M. altirostris* and *Naja naja*. In addition, a pool of PLA_2_ from *M. altirostris* venom (P4 from gel filtration) was used as a positive control (Figure 3A,D). Analysis of the concentration-dependent effect of *M. altirostris* (1–10 µg), *N. naja* (1–10 µg) crude venoms, and pooled PLA_2_ (0.1–3 µg) were used to assess 3 µg of venoms and 0.3 µg of pooled PLA_2_ as a suitable challenge dose (Figure 3A,D). Neither mAb 3B2 nor 1E8 were 100% efficient in neutralizing the PLA_2_ activity of 3 µg of *M. altirostris* venom (Figure 3B).

However, the mixture containing the same amount of each mAb enhanced the neutralizing capability (Figure 3B), further pointing to a synergistic effect in the reactivity of the mAb mixture. Similarly, mAbs 3B2 and 1E8 neutralized the enzymatic PLA_2_ activity of *N. naja* venom (Figure 3C) and pooled PLA_2_ (Figure 3E).

**Figure 4:**
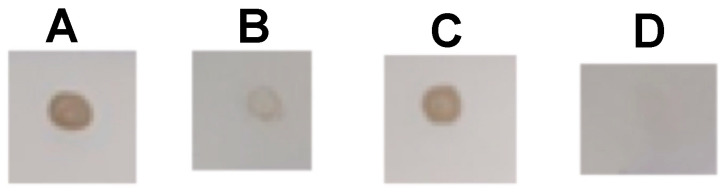
Dot blot of the *M. altirostris* venom against the monoclonal antibodies 1E8 (**A**,**B**) and 3B2 (**C**,**D**). In (**A**,**C**), native venom, (**B**,**D**) denatured venom: boiled for 5 min with 5% 2-mercaptoethanol.

**Figure 5:**
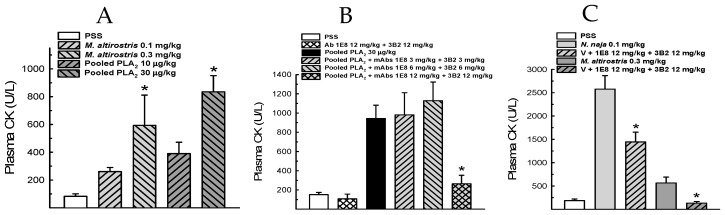
Effect of mAbs on myotoxicity. (**A**) shows the myotoxic effect of *M. altirostris* and pooled PLA_2_ (P4) in mice (*n* = 4–8). (**B**) the antagonism of the higher dose of immunoglobulins from clones 1E8 and 3B2 against pooled PLA_2_ (*n* = 4). (**C**) displays the antagonism of this same dose against *N. naja* and *M. altirostris* crude venoms. (*n* = 4). Data express mean ± SEM. * *p* < 0.05 vs. PSS (**A**) and * *p* < 0.05 vs. related venom in (**B**,**C**).

Each antibody was submitted to check its capability to recognize the native or denatured *M. altirostris* venom. Dot blot results showed that both mAbs were more effective against the native venom (Figure 4). These results point to the presence of conserved epitopes as the structural basis for the capability of mAbs 3B2 and 1E8 binding and inhibition of the enzymatic activity of PLA_2_ molecules. These results strongly suggest the implications of conformational structure to the epitopes for mAb 1E8 and 3B2. The synergic effect led to the inhibition of PLA_2_ molecules from phylogenetically distant elapid snakes (Figure 3C). Combined, these antibodies also neutralize PLA_2_ myotoxicity in vivo (Figure 5). The *M. altirostris* venom increased the plasma CK activity dose-dependently (Figure 5A).

Monoclonals 1E8 and 3B2 synergically neutralized this activity when pre-incubated with the venom. Similarly, the monoclonal antibodies neutralized the myotoxic activity of the pool of PLA_2_ (P4—Figure 5B). Regarding *N. naja* venom, which showed higher myotoxic activity than *M. altirostris* venom (Figure 5C), the mixture of monoclonal antibodies 1E8 and 3B2 partially antagonized this activity.

**Figure 6:**
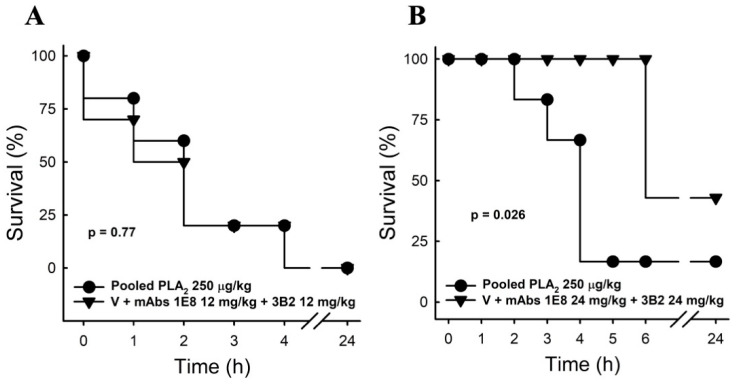
Survival curve. Mice were injected with pooled PLA_2_ alone or premixed for 15 min with immunoglobulins from clones 1E8 and 3B2 (i.p.) 12 mg/kg (**A**) or 24 mg/kg (**B**), and survival was observed for 24 h (*n* = 6–7). Data express the percentage of live mice, and the *p*-value represents the Mantel–Cox test analysis.

We then evaluated the capacity of these antibodies to neutralize the lethal activity of PLA_2_s. Since *M. altirostris* whole venom presents a high lethality also due to 3FTxs, we used the fraction P4 to evaluate the ability of mAbs 1E8 and 3B2 to neutralize the lethality of PLA_2_. To this end, we pre-incubated mAbs 1E8 and 3B2 (12 or 24 mg/kg each) with 1.5 × DL_50_ of the pooled PLA_2_s in fraction P4. The combination of both mAbs (24 mg/kg each) decreased the lethality of the pooled PLA_2_, thereby increasing by 50% the survival time of all treated animals and protecting more than 60% from death (Figure 6).

## 3. Discussion

Since the first experimental snake antivenom was raised in pigeons against the venom from *Sistrurus catenatus* Sewall, 1887, antivenom technology (essentially using immunization of large mammals with a mixture of crude venoms as antigen) has remained relatively unchanged for most of the 20th century [52]. Only recently, new therapeutic monoclonal antibodies or fragments of antibodies for antagonizing the effects of envenomation caused by spiders, scorpions, and snakes have been reported [48,52,53,54,55,56,57,58,59]. Monoclonal and recombinant antibodies have demonstrated effective neutralization activity of low-complexity venoms [59]. In this respect, El Ayeb and Rochat (1988) first generated monoclonal antibodies specific for the *Androctonus australis* AaH2 toxin, which exhibited neutralizing activity [60]. Licea et al. [61] also characterized a monoclonal antibody that neutralized *Centruroides noxius* venom on the ratio of 6 ug F(ab’)_2_ per ug of dose of venom. These approaches have been improving, and new applications in the production of antivenoms are emerging, like the production of humanized-camel single-domain antibodies, scFvs_,_ or recombinant human monoclonals obtained by phage display, among others [53,62,63]. Our present work aimed to generate monoclonal antibodies with broad specificity toward lethal toxins from *Micrurus altirostris* venom.

Micrurus venoms are not readily available; since these snakes have an ophiophagus diet, they are hardly maintained in captivity. In addition, due to their small size and small gland, they produce a low amount of venom in each extraction, thus hindering the production of anti-elapidic serum [15]. In that sense, obtaining mABs is an important strategy to increase antibody titles. *Micrurus* venoms are relatively simple regarding the class of lethal toxin families, which comprise mainly 3FTXs and PLA_2_ molecules [25,29,39]. Likewise, the lethality of *M. altirostris* venom is triggered by the same protein classes. High toxicity (LD_50_ of 3.3 and 2.9 µg/mouse) was found on the venom fractions enriched with PLA_2s_ (P4) and 3FTXs (P8), respectively (Table 1). Regarding the PLA_2_, the venomics analysis identified eight proteoforms (13.7% of total venom protein), and among them five were assigned for the transcripts F5CPF1, F5CPF0, and AED89576, pointing to the presence of isoforms. The transcript F5CPF0 was identified in RP-HPLC peak 20, which was specifically recognized by mAb 3B2. F5CPF1 and AED89576 corresponding to PLA_2_s, which share a conserved structural epitope recognized by mAb 1E8 (RP-HPLC peaks 21–25).

It is worth mentioning that F5CPF1 and AED89576 showed close phylogenetic relationships with MmipPLA_2_, the most abundant PLA_2_ of *M. mipartitus* venom (accounting for nearly 10% of the total venom protein) [41]. Functional characterization of MmipPLA_2_ revealed a toxin with low enzymatic activity and myotoxicity but high lethality in mice [40]. A similar functional pattern was found for fraction P4 isolated from *M. altirostris* venom (Table 1, Figure 3D), highlighting the importance of developing inhibitors for each type of PLA_2_. The monoclonal antibodies produced here could inhibit the catalytic activity and in vivo myotoxicity of *M. altirostris, N. naja* venoms, and pooled PLA_2_. Furthermore, these antibodies increased the survival time of all envenomed animals by 50% by protecting more than 60% from death (Figure 5). It is worth emphasizing that the protocol used for the evaluation of survival uses premixed antibodies/venom proportions, which implies a limitation for a clinical application that must be observed in further experiments.

The results indicate the involvement of two epitopes in this process, both antibodies inhibiting enzymatic and toxic activities. Moreover, these anti-PLA_2_ mAbs exhibited paraspecificity against PLA_2_ molecules from *N. naja* venom, suggesting the conservation of neutralizing paraspecific structural epitopes across the Elapidae family. Both mABs 1E8 and 3B2 recognized structural PLA_2_ epitopes, demonstrating that despite differences in primary structure, these toxin classes present high immunological similarity, corroborating the existence of antibodies sharing broad neutralization of family-specific snake venom toxic proteins. Chavanayarn et al., 2012 [62], produced Humanized Single Domain Antibodies against the PLA2s from *Naja kaouthia*, inhibiting enzymatic activity. By modeling and docking analysis, they suggested that these antibodies’ CDR loops are inserted into PLA_2_ catalytic grooves. We aligned the sequence from *Naja naja*, *Naja atra*, and *M. altirostris* PLA_2_s and verified manually that the residues from the calcium binding site and lipid binding site are shared between these molecules (Figure S3). Our data indicate that a similar mechanism may be involved. Future structural analyses are necessary to confirm this hypothesis.

Our results suggest the applicability of mAb 1E8 and 3B2 to supplement current conventional antivenoms to improve the neutralization capacity of the antisera. For example, the Brazilian coral snake antivenom lacks neutralization activity towards the venom of *M. altirostris* [43,44,45]. The antivenomic analysis revealed low immunoreactivity for some toxins from *M. altirostris* venom, particularly PLA_2_ F5CPF0, which entirely escaped immunodepletion by coral snake antivenom [16]. However, this toxin was neutralized by monoclonal antibodies described in this work. Our results demonstrate potential applicability, especially to neutralize the lethal activity of coral snakes from venoms enriched with PLA_2_, including *M. fulvius*, *M. moscatensis*, and *M. dumerilli*, which have 58%, 55%, and 52% of PLA_2_, respectively [29]. Furthermore, other preparations may improve antivenoms’ effectiveness, such as combining polyclonal and monoclonal antibodies with neostigmine, a cholinesterase inhibitor that has been applied to overcome the effects of α-neurotoxins [64,65]. Future studies must confirm if this supplementation to antielapidic sera will produce more protection. Additionally, new immunization strategies were recently driven by the vaccine market and consequently brought a lot of innovation to this area; these include DNA or mRNA vaccines, among others [66,67], and they can be used to develop new antisera.

Two divergent patterns of coral snakes’ venom composition, 3FTx-rich and PLA2-rich, have been revealed by proteomics studies [20,28,29]. Our work indicates the potential use of monoclonal antibodies 1E8 and 3B2 for neutralizing the PLA_2_ molecules of PLA_2_-rich venoms of coral snakes from Central and North America.

## 4. Conclusions

Our present work aimed to generate monoclonal antibodies with broad specificity toward lethal toxins from *Elapidae* venom. The toxicity was analyzed using the toxicovenomics approach, and all PLA_2_ molecules were recognized by the pair of monoclonal antibodies, pointing to a synergistic effect in the reactivity. These antibodies increased by 50% the survival time of all envenomed animals by protecting more than 60% from death. They inhibited PLA_2_ catalytic activity and showed paraspecific neutralization against the myotoxicity from the lethal effect of *Micrurus altirrostris* and *Naja naja* venoms’ PLA_2_s. Epitope conservation among venom PLA_2_ molecules suggests the conservation of neutralizing paraspecific structural epitopes across the Elapidae family and the possibility of generating pan-PLA_2_ neutralizing antibodies.

## 5. Materials and Methods

### 5.1. Materials

Crude venom of *M. altirostris* was pooled from adult specimens from Southern Brazil and kept in the serpentarium of Núcleo de Ofiologia de Porto Alegre (NOPA), Rio Grande do Sul, Brazil, under the register of IBAMA no. 1/43/1999/000764-9, and Sisgen code A9F3694. Crude venoms were lyophilized and stored at −20 °C until use. *Naja naja* (batch no. V-9125) venom was purchased from Sigma Chemical Co (Saint Louis, MS, USA). Creatine kinase (CK) activity was determined using a CK NAC^®^ kit from Bioclin (Belo Horizonte, MG, Brazil). Male Swiss mice (20.0 ± 2.0 g) used for the study received water and food ad libitum and were kept under a natural light cycle. Animal handling was conducted in agreement with Ethical Principles in Animal Research and approved by the Ethical Committee for Animal Experimentation from Universidade Federal do Rio de Janeiro and from Instituto Vital Brazil (CEUA-UFRJ: no. 01200.001568/2013-87; CEUA-IVB: no. 001/2016).

### 5.2. ELISA

Microtiter plates were coated with the proteins eluted (1 µg/well) in the different reverse-phase HPLC fractions isolated as described [16]. Plates were blocked with 3% bovine serum albumin (BSA) before incubating with monoclonal antibodies for 1 h at room temperature. Plates were then washed with PBS with 0.05% Tween 20, and antigen-antibody complexes were detected using the peroxidase-conjugated anti-mouse IgG/ortho-phenylenediamine colorimetric method, following the manufacturer’s (Sigma, Saint Louis, MS, USA) instructions. Absorbance was recorded at 492 nm.

### 5.3. M. altirostris Venom Fractionation and In Vivo Toxicity Analysis (LD_50_)

Crude venom (20 mg in Tris-HCl pH 7.5) was fractionated by TSK^®^-gel size-exclusion chromatography and yielded eight fractions. To determine the median lethal dose (LD_50_), we followed the protocol described by World Health Organization [68]. Briefly, 0.5 mL of the whole venom and the manually collected fractions from the TSK^®^ column (in PBS) were intraperitoneally administered to 18–22 g male Swiss mice. Six mice were used for each dose (four dose levels per test), and deaths occurring within 24 h were recorded. The LD_50_ was calculated by probit analysis, using the formula LD_50_ = LD_100_ − ∑ (a × b)/n, where: n = total number of animals in the group; a = the difference between two successive doses of administered extract/substance; b = the average number of dead animals in two successive doses; and LD_100_ = lethal dose causing 100% death of all test animals [60].

The Identification of venom components eluted in the gel-filtration chromatographic fractions was performed by chromatogram comparison with the venomic characterization previously described in [16]. Briefly, the venom or gel-filtration fractions were separated by reverse-phase HPLC using a Shimadzu chromatograph and a Teknokroma Europa C18 (0.4 cm × 25 cm, five μm particle size, 300 Å pore size) column eluted at 1 mL/min with a linear gradient of (5–70%) of 0.1% TFA in water (solution A) and 0.1% TFA in acetonitrile (solution B). The absorbance was recorded at 214 nm, and peaks were manually collected.

### 5.4. Production and Purification of Monoclonal Antibodies (mAbs)

Monoclonal antibodies were produced as described by [69] with modifications. Briefly, BALB/c mice were inoculated with fraction five (P5) of *M. altirostris* venom. The P5 was chosen because it presents the two main families of toxins in a similar ratio. Furthermore, the low toxicity allowed increasing the amount of toxins from P5 up to 30 μg per animal in the immunization process. Then, popliteal lymph node cells from BALB/c mice were fused with SP2-O cells (2:1) using polyethylene glycol 4000 (Merck). Hybrid cells were selected in RPMI 1640 medium plus 3% HAT (hypoxanthine 10 mM, aminopterin 40 mM and thymidine 1.6 mM) (GibcoBRL) containing 10% FCS (GibcoBRL) at 37 °C and 5% CO_2_. The supernatant was screened for toxin-specific antibodies by ELISA, as described above. Antibody-secreting cells were expanded and cloned twice at limiting dilution. The mAbs contained in the culture supernatants were purified by affinity chromatography on protein-A Sepharose (Pharmacia) equilibrated in borate saline buffer (BSB), pH 8.5. The proteins were eluted in 0.2 glycine/HCl buffer, 0.15 M NaCl, pH 2.8, and dialyzed in BSB.

### 5.5. Monoclonal-Based Antivenomics

We employed the second-generation affinity chromatography-based antivenomic protocol [70]. To this end, 400 μL of NHS-activated Sepharose 4 Fast Flow (GE Healthcare) were packed in a column and washed with 10–15 matrix volumes of cold 1 mM HCl, followed by two matrix volumes of coupling buffer (0.2 M NaHCO_3_, 0.5 M NaCl, pH 8.3) to adjust the pH of the column between 7.0–8.0. Then, 1 mg of monoclonal antibody in 300 µL of coupling buffer was incubated with the activated matrix in an orbital shaker overnight at 4 °C. Non-saturated reactive NHS-matrix functional groups were blocked with 400 μL of 0.1 M Tris-HCl, pH 8.5, overnight at 4 °C in an orbital shaker. The affinity columns were 6× washed alternating at high (0.1 M acetate buffer, 0.5 M NaCl, pH 4.0–5.0) and low pH (0.1 M Tris-HCl buffer, pH 8.5). Before incubating with crude venom, the affinity matrix was equilibrated with five matrix volumes of binding buffer (PBS). For the immunoaffinity assay, 300 μg of *M. altirostris* venom were incubated 3 h with the matrix at room temperature (~25 °C) in an orbital shaker. As a specificity control, 300 μL of Sepharose 4 Fast Flow matrix was incubated with venom, and the mock column was developed in parallel to the immunoaffinity column. After eluting the non-binding fraction, the affinity column was washed twice with PBS. The immunocaptured venom proteins were eluted with 2.5 matrix volumes of elution buffer (0.1 M glycine-HCl, pH 2.0) and neutralized with neutralization buffer (1 M Tris-HCl, pH 9.0). The non-retained and the immunocaptured venom fractions were fractionated by reverse-phase HPLC and identified by overlap with the chromatogram reported in the previous homologous venom proteome characterization study [16].

### 5.6. Dot Blot

For the dot blot, denatured venom, after boiling (5 min) with 5% 2-mercaptoethanol, or native *M. altirostris* crude venoms were applied directly to membranes (strips) of nitrocellulose at a concentration of 10 μg/μL. The nitrocellulose-sensitized membranes were incubated for 2 h at room temperature with a blocking solution (5% skim milk in Tris-saline) under constant stirring. At the end of the reactive group blocking, the membranes were washed for 3 min each with Tris-saline. Then the membranes were incubated with monoclonal antibodies 1E8 or 3B2 for 2 h under constant stirring at room temperature. Membranes were subjected to a new wash cycle with Tris-saline and incubated for 2 h under continuous stirring with peroxidase-labeled mouse anti-IgG sheep antibody diluted 1:5000 in Tris-saline containing 1% skim milk. The reaction was developed with the chromogenic substrate, 0.05% 4-chloro-1-naphthol in 15% methanol, and color was developed by adding 0.03% H_2_O_2_.

### 5.7. Phospholipase Activity In Vitro

Phospholipase A_2_ activity was assessed by the turbidimetric assay [71] adapted by [72]. The stock substrate was prepared by shaking one chicken egg yolk in 150 mM NaCl to a final volume of 100 mL. This substrate was stored at 4 °C until used. For the PLA_2_ activity assay, a 1 mL substrate solution containing 0.63 mL of a 10% dilution of the egg yolk stock suspension, 150 mM NaCl, 10 mM CaCl_2_, 0.01% taurocholic acid, and 5.0 mM Tris-HCl (pH 7.4), was freshly prepared. Then, 0.13 mL of this solution was added to each microtiter plate well containing either 0.07 mL of saline, venom alone, or venom/mAb mixture. The ELISA microplate was kept at 37 °C during the procedure. The reaction was started by adding venom (1–30 µg/mL) of *Micrurus altirostris*, *M. corallinus*, *Naja naja*, or a pool of PLA_2_ purified from *M. altirostris* venom (0.1–3 µg/mL) into the yolk solution. The concentration of 3 µg/mL of venoms and 0.3 µg/mL of pooled PLA_2_ was chosen to challenge the mAb 1E8 and 3B2 (60–480 µg/mL) alone or mixed; the absorbance of the solutions was measured at 925 nm every 5 min for 30 min for crude venom and 60 min for pooled PLA_2_. Data were expressed as absorbance reduction and percentage venom activity.

### 5.8. Myotoxic Activity In Vivo

We evaluated the myotoxicity of *M. altirostris*, *N. naja* venoms, and P4 (pooled PLA_2_) by measuring the increase of plasma CK activity induced by intra-muscular (i.m.) injection of venom alone or associated with mAb (3B2 and 1E8). The venom was dissolved in physiological saline solution (PSS) to a final volume of 0.16 mL and injected into the rear thigh of mice as described previously [73,74]. Negative controls consisted of mice injected with the same volume of PSS. To evaluate the antimyotoxic activity of the mAb (3B2 and 1E8), the protocol used was pre-incubation. Before injection, the venoms were dissolved in PSS and incubated with mAb 3B2, 1E8 (1:1) for 15 min at room temperature. The doses used here were based on the dose-response study, from which we chose the most effective ones. Enzyme activity was expressed as international units, where 1 U is the amount that catalyzes the transformation of 1.0 µmol of the substrate at 25 °C per minute.

### 5.9. Inhibition of Lethality by mAbs

To evaluate the lethality effect of pooled PLA_2_ (P4) and the protection by mAb (3B2 and 1E8) in mice (groups of 6–7), we used the method as described in [75] with modification. Briefly, 1.5 LD_50_ (5 µg/animal or 250 µg/kg) of P4 and the mAb (12 and 24 mg/kg of each mAb) were pre-incubated for 15 min and then administered by intraperitoneal (i.p.) injection. Mice were observed for 24 h. Each hour the number of survivors was counted.

### 5.10. Statistical Analysis

Data were expressed as mean ± SEM. The number of experiments performed is provided in the legends of the figures, for instance, as (*n* = 5), meaning there were five samples analyzed in one experiment. One-Way Analysis of Variance (ANOVA) was used to compare groups with one variable, followed by Dunnett’s post-hoc test. For two variables, Two-Way Analysis of Variance (ANOVA) was used, followed by Bonferroni’s post-hoc test. The *p*-value < 0.05 was used to indicate a significant difference between the means. The software GraphPad Prism version 5.01 was used to provide statistical analysis. Graphs were made using the Sigmaplot program version 10.0.

## Figures and Tables

**Table 1:** An overview of lethal toxicity of *M. altirostris* venom compounds. The venom was fractionated by TSK-gel filtration (Figure 1), and the samples peak 1 to peak 8 (P1–P8) were identified by chromatogram comparison of RP-HPLC analyses (Figures S1 and S2). For the samples that showed lethality with less than 8 μg/mice (~20 g), determined by six mice/dose, the LD_50_ is indicated next to the sample number.

Gel Filtration Sample (LD_50_ μg/Animal)	RP-HPLC Analyses	Protein Identification *
P1	31	LAO
P2	31	LAO
P3	18, 19, 20, 21, 22	PLA_2_
P4 (3.3)	17 20, 21, 23–25	3FTx (10.1%) PLA_2_
P5	30 1–5, 7, 8, 10, 13, 16 20, 21, 22, 23	LIPA 3FTx PLA_2_
P6 (6.92)	1–9, 13 21	3FTx PLA_2_ (6.3%)
P7 (7.07)	1–9, 13 21	3FTx PLA_2_ (6.3%)
P8 (2.97)	2, 3, 5, 8, 1.	3FTx

* LAO—L-amino-oxidase; PLA_2_ phospholipase A2; 3FTx—three-finger toxin.

**Table 2:** Overview of the target of monoclonal antibodies. The specificity showed by each monoclonal antibody produced against 3FTx and PLA_2_ molecules. The reactivity was measured by ELISA (Supplementary Materials).

Monoclonal	Target	
	3FTx	PLA_2_
1E8		++ ^1^
2G2	++	
2B1	++	
3B2		+++
4B3	+++	

^1^ The increase of absorbance quantified as follow ++ DO ≤ 2 > 1, +++ DO > 2.

## Data Availability

Not applicable.

## Supplementary Materials

The following supporting information can be downloaded at: https://www.mdpi.com/article/10.3390/toxins15010015/s1, Figure S1:The chromatograms separation of *M. altirostris* venom by RP-HPLC showing the peaks that were previously identified [15]; Figure S2: The chromatograms separation of TSK fractions by RP-HPLC; Figure S3: Alignment of phospholipases A2.
