# Supplementary material for: Monoclonal-Based Antivenomics Reveals Conserved Neutralizing Epitopes in Type I PLA2 Molecules from Coral Snakes
Source: Toxins (Basel). 2022 Dec 26;15(1):15. doi: 10.3390/toxins15010015 (PMC9863321; doi:10.3390/toxins15010015)
Supplement: Supplementary file 1 [file toxins-15-00015-s001.zip › toxins-2060284-supplementary.pdf]

Supplementary Material: Monoclonal-Based Antivenomics Reveals Conserved Neutralizing Epitopes in Type I PLA_2 Molecules from Coral Snakes

Carlos Corrêa-Netto, Marcelo A. Strauch, Marcos Monteiro-Machado, Ricardo Teixeira-Araújo, Juliana Guzzo Fonseca, Moema Leitão-Araújo, Maria Lúcia Machado-Alves, Libia Sanz, Juan J. Calvete, Paulo A. Melo and Russolina Benedeta Zingali

Each fraction of TSK-gel filtration (Article Figure 1) was identified by chromatograms comparison and retention time of each protein (Figure S1 and S2). The chromatograms were aligned and the fourth fraction of gel filtration (P4) was chosen as example to illustrate the identification by comparison of the retention time of samples (Figure S2). Next e protein identifications of each fraction from TSK-gel filtration (P1-P8) are indicated.

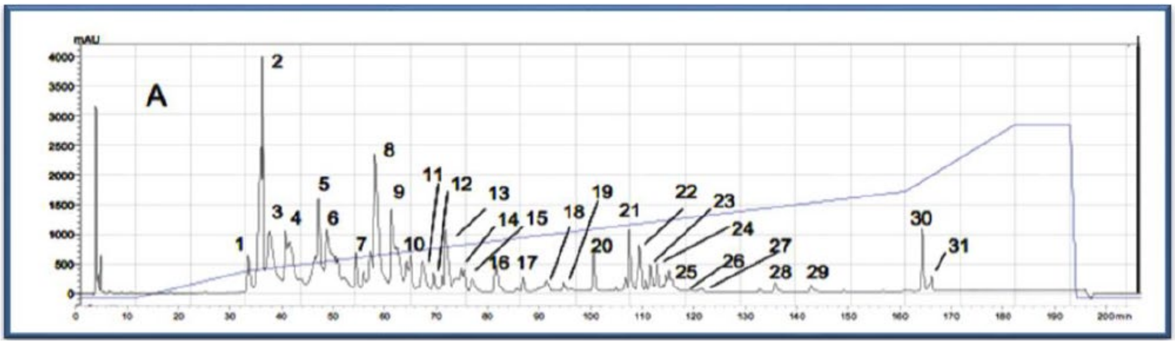

Figure S1: The chromatograms separation of *M. altirostris* venom by RP-HPLC showing the peaks that were previously identified [15]. Identification of gel filtration fractions was achieved by their elution in the RP-HPLC. Each fraction from the TSK-gel filtration (Figure 1) was compared with the chromatogram of total venom, following the same conditions, and retention time of each peak (A) was aligned with a standard migration of venom proteins. Thus, the fractions of P1-P8 were identified.

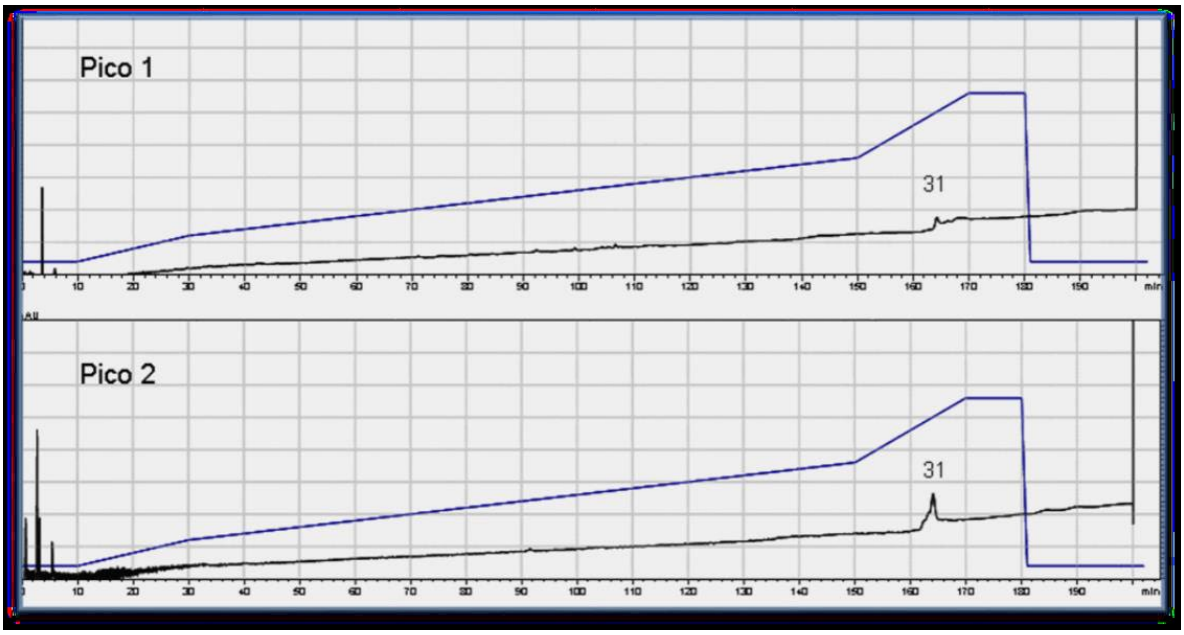

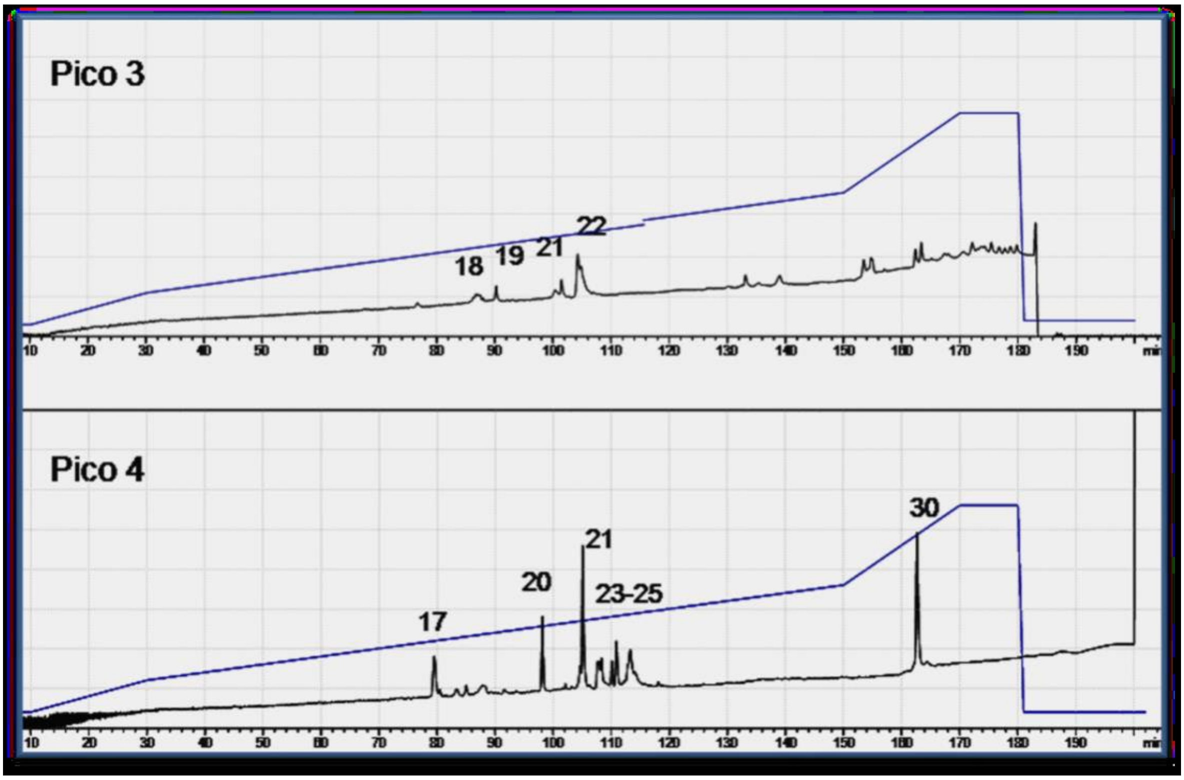
Pico 3
18
19
21
22
Pico 4
17
20
21
23-25
30
10 20 30 40 50 60 70 80 90 100 110 120 130 140 150 160 170 180 190

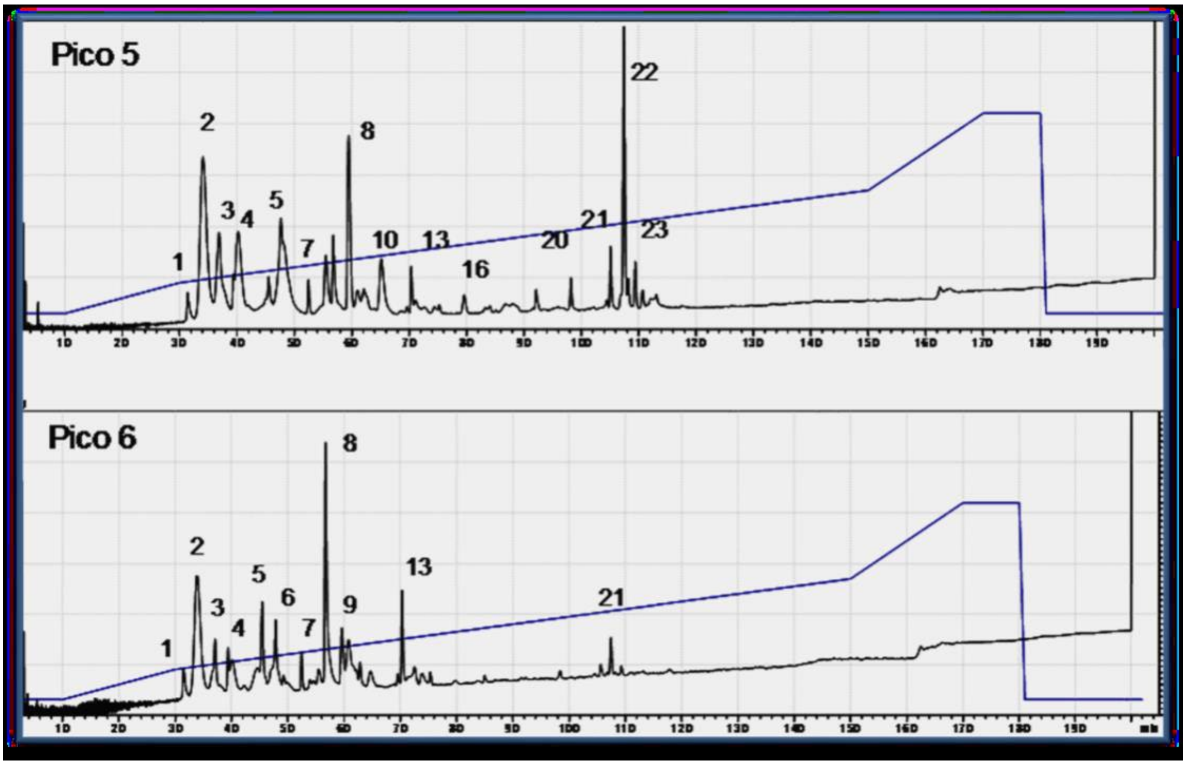
Pico 5
1
2
3
4
5
7
8
10
13
16
20
21
22
23
Pico 6
1
2
3
4
5
6
7
8
9
13
21
10 20 30 40 50 60 70 80 90 100 110 120 130 140 150 160 170 180 190

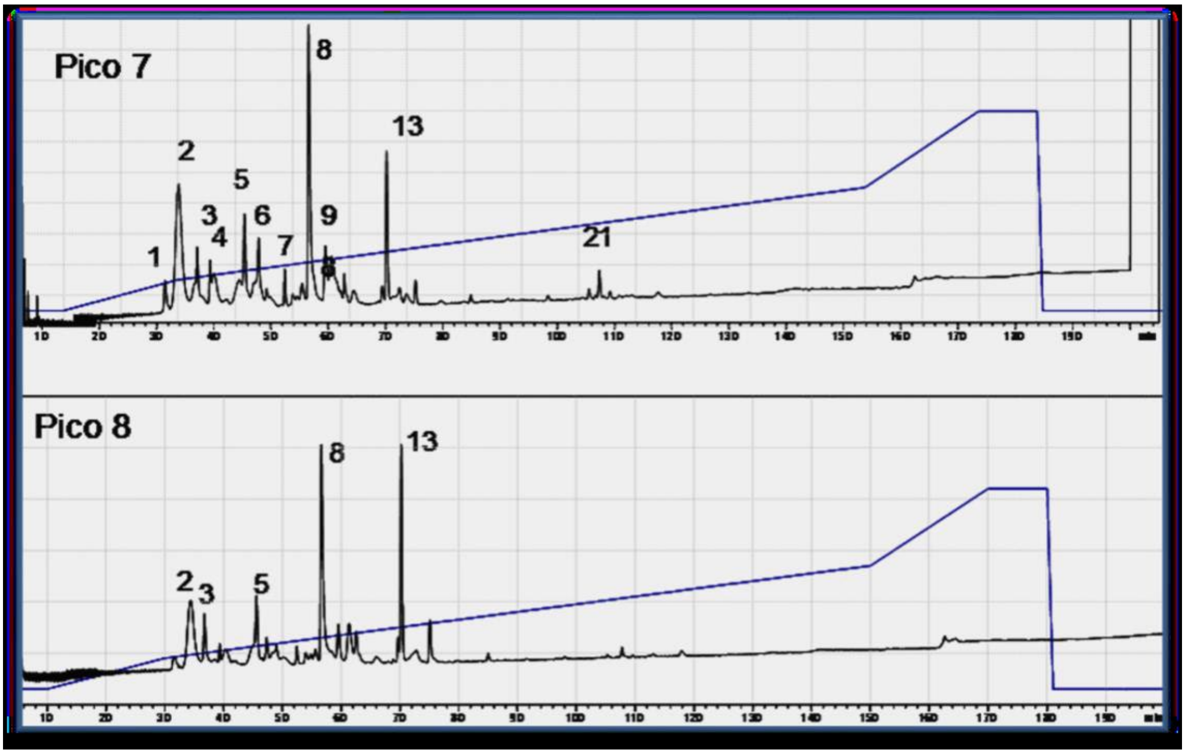

Figure S2: The chromatograms separation of TSK fractions by RP-HPLC.

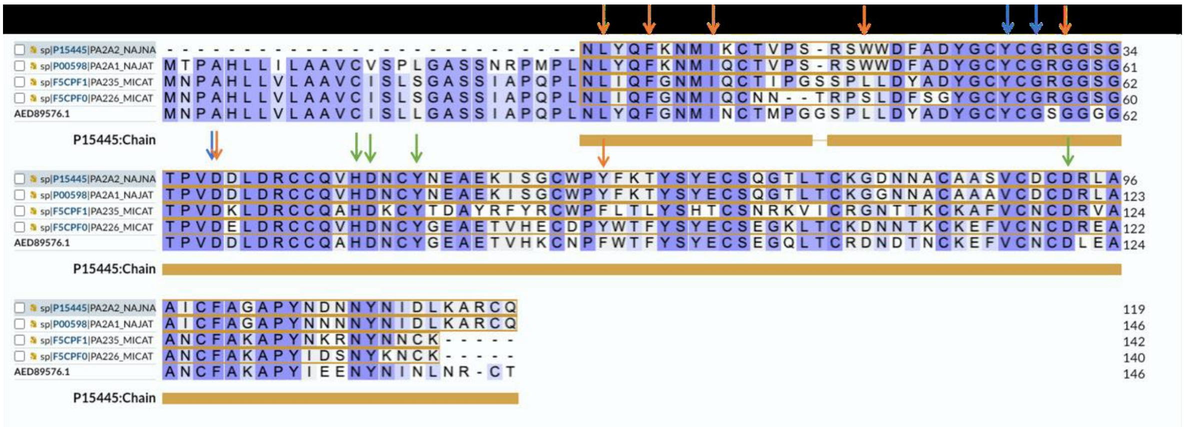

Figure S3: Alignment of phospholipases A2. Phospholipases from *Naja naja* P15445; *Naja atra* P00598, *M. altirostris* F5CPF1, F5CPF0, and AED89576.1 (Genebank) were aligned using Clustal platform at UNIPROT site (https://www.uniprot.org/ accessed 12/12/2022). Dark purple means identical amino acid residues, and light purple means similar amino acids. Arrows indicate residues that may be related to antibodies recognition, orange active site (H47, D48, Y51, and D93), blue Ca2+ binding site (Y27, G29, G31, and D48), and green phospholipid binding (L2, F5, I9, W19, F21, A22, G31, and Y63).
